# Performance Improvement during Attitude Motion of a Vehicle Using Aerodynamic-Surface-Based Anti-Jerk Predictive Controller

**DOI:** 10.3390/s23125714

**Published:** 2023-06-19

**Authors:** Ejaz Ahmad, Iljoong Youn

**Affiliations:** Department of Mechanical and Aerospace Engineering, Gyeongsang National University, ReCAPT, Jinju 52828, Republic of Korea; ejaz@gnu.ac.kr

**Keywords:** anti-jerk control, predictive control, braking, half-car model, attitude motion tacking, lane-change maneuver

## Abstract

This study presents the effectiveness of an anti-jerk predictive controller (AJPC) based on active aerodynamic surfaces to handle upcoming road maneuvers and enhance vehicle ride quality by mitigating external jerks operating on the body of the vehicle. In order to eliminate body jerk and improve ride comfort and road holding during turning, accelerating, or braking, the proposed control approach assists the vehicle in tracking the desired attitude position and achieving a realistic operation of the active aerodynamic surface. Vehicle speed and upcoming road data are used to calculate the desired attitude (roll or pitch) angles. The simulation results are performed for AJPC and predictive control strategies without jerk using MATLAB. The simulation results and comparison based on root-mean-square (rms) values show that compared to the predictive control strategy without jerk, the proposed control strategy significantly reduces the effects of vehicle body jerks transmitted to the passengers, improving ride comfort without degrading vehicle handling at the cost of slow desired angle tracking.

## 1. Introduction

The advancements in the design of vehicle geometry to enhance vehicle ride comfort and road-holding capability over the past few decades have significantly contributed to the automotive industry [1,2,3,4]. The uncomfortable vibrations that the vehicle body transmits to the passengers have an impact on ride comfort. To improve the tire’s traction on the road, road holding refers to lowering oscillations in the typical wheel load. Though these two are the essential components of vehicle ride performance, there is always a trade-off between ride comfort and road-holding capability. Therefore, an appropriate control system framework that can easily address this aspect is essential to enhance ride performance. In [5], a hybrid fuzzy controller was used to reduce the trade-off between ride comfort and road holding. In [6], an optimal trade-off was achieved using a passive–active-based control approach. As reported in [7], we have used an active suspension control strategy integrated with an active seat to improve ride comfort and road-holding capability. The researchers in [8] reported a substantial improvement in ride comfort and road-holding capability considering various road conditions. In [9], an approximation-free control for active suspension system was used to improve the ride comfort. To address the trade-off between these two components of ride performance, this research was mostly based on active suspension or semi-active suspension systems; however, recent research on the applications of aerodynamic-surface-based control strategies has gained significant impetus to improve vehicle performance.

Active aerodynamic-surface-based control strategies can significantly increase negative lift force with increased vehicle speed, effectively improving ride performance. Therefore, applications of active aerodynamic surfaces (AASs) installed on the vehicle sprung mass has attracted many researchers’ interest. For example, Savkoor [10,11,12] published early primary research on the applications of AAS, using several control strategies to reduce the heave and pitch angle of a truck cabin. Active aerodynamic control (AAC) strategies are also effective in improving the lateral performance of a road vehicle. In [13], Doniselli et al. investigated how aerodynamics affected a high-speed car’s ride quality on a randomly contoured route. The recent research on sports cars by [14,15,16,17,18] used various control approaches to investigate the applications of AAS to improve ride comfort. To improve the vehicle’s handling, AACs have been used to adjust aerodynamic surfaces and provide a range of negative lift forces [19,20,21]. In our earlier research [22,23], we examined how aerodynamic surfaces could produce a negative lift force to enhance a vehicle’s ride quality while considering pitch and roll dynamics. Though these pieces of research effectively improve ride performance, their performance depends on AAS’s idealistic independent operation. The major challenge with vehicles equipped with AAS is the realistic motion of aerodynamic surfaces installed on the unsprung mass. Due to high speed, the sharp movement of aerodynamic surfaces will result in high vehicle body jerk and acceleration, severely affecting passenger ride comfort. Therefore, an appropriate solution to reduce the adverse impact of vehicle body jerk on passengers to ensure better ride comfort and road-holding capability is aimed toward an anti-jerk control strategy.

As discussed by [24], the term “jerk” is considered a better performance parameter for measuring ride comfort than acceleration and is widely used in engineering applications. For example, it has been considered as a ride comfort parameter for amusement rides [25,26,27], elevators [28], ships [29], and buses [30]. Jerk is considered an important passenger ride discomfort parameter in vehicles and is extensively discussed in automotive engineering. Anti-jerk controllers have been commonly employed in electric vehicles to reduce longitudinal jerk to enhance ride comfort and drivability [31,32]. Hence, anti-jerk control strategies have inspired many researchers to enhance ride comfort by reducing longitudinal jerk produced during the starting of electric vehicles. A non-linear predictive-based anti-jerk cruise controller model was developed in [33] for electric vehicles to reduce longitudinal jerk and improve passenger ride comfort. In [34], a predictive anti-jerk controller model was developed to overcome the trade-off between ride comfort and vehicle handling. In [35], an anti-jerk controller was developed for a hybrid electric vehicle to reduce the jerk produced during clutch start. To track the intended velocity with the least jerk and improved road safety, ref. [36] employed a linear-quadratic-based anti-jerk controller. Ref. [37] utilized a low-jerk suspension control technology to improve ride comfort. A backlash-based anti-jerk controller was employed in [38] to reduce jerk during clutch engagement. However, these anti-jerk control strategies minimize longitudinal jerk to improve ride comfort, while the research on improving vehicle performance during lateral or vertical motion is very limited, and early efforts by Hrovat and Hubbard [39,40] implemented an anti-jerk control strategy to enhance ride comfort during vertical motion of a quarter-car model. Where an augmented performance index was introduced to include the jerk rms term in addition to other outputs to improve ride comfort, their results for the one-degree-of-freedom (DOF) quarter-car model showed a reduction in rms jerk at the cost of an increase in the rms of heaving acceleration, tire deflection, and rattle space. In [41], they further investigated the application of an optimal anti-jerk controller for a two-DOF quarter-car model. They predicted that a maximum reduction in rms jerk can be obtained at a cost of a modest increase of 23% in rattle space and a significant increase of 127% in tire deflection. Hence, such significant improvements in ride quality can be achievable at the penalty of vehicle handling. In [42], using a semi-active suspension system, we have implemented a preview-based anti-jerk control strategy to improve ride comfort without degrading the road-holding capability under different road conditions. Despite the exciting results, to the researcher’s knowledge, the previous anti-jerk methodologies only focus on enhancing ride comfort using conventional active or semi-active suspension systems. Moreover, the high speed of vehicles can also limit the application of these conventional methodologies. Therefore, in [43], we have implemented an active aerodynamic-based anti-jerk control strategy on a half-car model to improve the vehicle performance under different road conditions, i.e., bump inputs and asphalt roads. However, load transfer effects during lateral motion are not considered, which greatly impacts passengers’ ride comfort during cornering, braking, or forward acceleration.

Leaning the vehicle body against the vehicle body forces during lateral or longitudinal motion is very useful to mitigate the load transfer effects to enhance ride comfort. For example, the authors of [44,45] used an anti-roll bar methodology to reduce the impact of load transfer during cornering. In [46,47], tilting control systems were developed to improve vehicle safety during cornering. In [48], we have designed a preview-based attitude controller to reduce the load transfer effect and track the desired roll and pitch position during cornering or forward acceleration to enhance ride quality. Similarly, in [7], using a conventional active suspension system, an attitude motion controller was developed for vehicles with active passenger seat systems to improve ride comfort and vehicle handling. In [23], we implemented an AAC strategy to tilt the vehicle body against centrifugal or inertial forces to track the desired roll or pitch position to enhance ride performance. This performance was achieved by the independent operation AAS, which is an idealistic approach. AASs are directly installed on the sprung mass of the vehicle; therefore, their movement also has a direct impact on vehicle body jerk, which is an important ride comfort parameter. This is because negative lift force or downforce generated by aerodynamic surfaces strictly depends upon vehicle speed and the angle of attack. Therefore, it is important to achieve realistic motion of aerodynamic surfaces to reduce vehicle body jerk during attitude motion.

Motivated by these perceptions, in this paper, a four-degrees-of-freedom half-car model equipped with aerodynamic surfaces is considered to explore the applications of aerodynamic-based anti-jerk optimal control strategy, which comprises a feed-forward control strategy in addition to a state feedback controller. The feed-forward control can anticipate the force required to track the desired attitude angle. The state feedback controller can adjust the force to minimize the tracking error. Our main goal is to achieve the realistic motion of aerodynamic surfaces to minimize vehicle body jerks without degrading the road-holding capability. Anti-jerk optimal control with known predicted information regarding the future road maneuver is proposed to enhance the ride comfort during cornering, braking, or accelerating. The difference between braking and cornering is that braking performance is dependent only on the vehicle’s speed, while cornering performance is dependent on the speed of the vehicle as well as the radius of the curvature. The proposed optimal predictive control strategy can generate anticipating actions against future road maneuvers. The following are the distinguished features that contribute to this work:

- A ubiquitous four-DOF half car equipped with AAS is presented as a case study.
- Information about future road maneuvers can be obtained by direct detection using sensors attached to the vehicle with a 0.3 s preview time.
- The desired roll or pitch angles are computed using vehicle speed, future road maneuvers, and the disturbance forces acting on the vehicle body.
- The proposed control scheme aims to improve ride performance by canceling external jerks.
- The simulation results are carried out using MATLAB to validate the effectiveness of the proposed anti-jerk predictive control strategy in terms of reducing the controlling jerk to achieve the smooth movement of AAS, to overcome the trade-off between ride comfort and road-holding at the cost of slow tracking.

The rest of the paper is organized as follows: In Section 2, the problem formulation is presented. Section 3 represents the proposed optimal anti-jerk control strategy, while Section 4 discusses the simulation results, followed by a conclusion with future recommendations.

## 2. Problem Formulation

### 2.1. Vehicle Model

**Figure 1:**
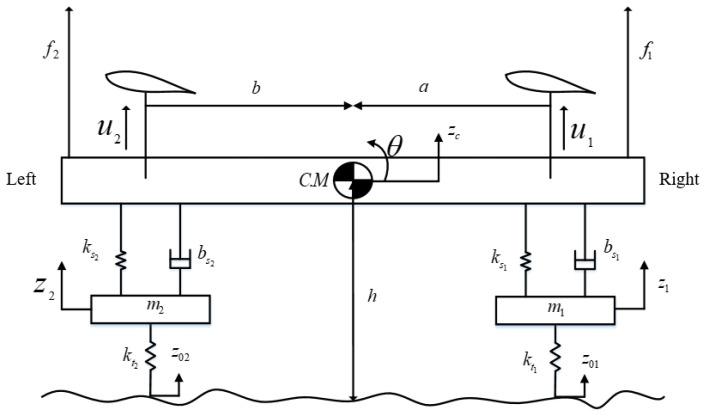
Four-DOF lateral half-car model with active aerodynamic surfaces.

A schematic diagram of a four-degrees-of-freedom half-car model is shown in Figure 1, which can be considered as a longitudinal model in a forward direction or a lateral model during cornering. The proposed model comprises two unsprung masses and one sprung mass. The unsprung mass consists of masses $m_{1}$ and $m_{2}$, a damper with damping coefficients $b_{s_{1}}$ and $b_{s_{2}}$, spring with stiffness coefficients $k_{s_{1}}$ and $k_{s_{2}}$, and tire stiffness coefficients $k_{t_{1}}$ and $k_{t_{2}}$ at the right and left side, respectively. In contrast to conventional active suspension systems, two aerodynamic surfaces mounted on the sprung mass provide the necessary negative lift forces, $u_{1}$ and $u_{2}$, to enhance ride comfort and road-holding capability. The hypothetical body forces, $f_{1}$ and $f_{2}$, act on the vehicle body during cornering, braking, or forward acceleration. The parameter values of the addressed model are shown in Table 1. The mathematical model is derived using Newtonian methods.

The equations of motion for the sprung mass acceleration and roll or pitch angle are given as follows:

(1) $$\begin{array}{cc} M{\ddot{z}}_{c} & =f_{l}+f_{r}+u_{1}+u_{2}+f_{1}+f_{2} \end{array}$$

(2) $$\begin{array}{cc} I\ddot{\theta } & =a\left(f_{r}+u_{1}+f_{1}\right)-b\left(f_{l}+u_{2}+f_{2}\right) \end{array}$$

where $Z_{c}$ is the sprung mass displacement, *M* is the sprung mass, θ is the attitude angle of the vehicle body, *I* is known as the moment of inertia, $f_{r}$ and $f_{l}$ are right and left side suspension forces given in Equations (3) and (4), respectively:

(3) $$\begin{array}{cc} f_{r} & =b_{s_{1}}\left({\dot{z}}_{1}-a\dot{\theta }-\dot{z_{c}}\right)+k_{s_{1}}\left(z_{1}-a\theta -z_{c}\right) \end{array}$$

(4) $$\begin{array}{cc} f_{l} & =b_{s_{2}}\left({\dot{z}}_{2}+b\dot{\theta }-\dot{z_{c}}\right)+k_{s_{2}}\left(z_{2}+b\theta -z_{c}\right) \end{array}$$

For the unsprung masses, the equations are given as:

(5) $$\begin{array}{c} m_{1}{\ddot{z}}_{1}=-\left(k_{t_{1}}\left(z_{1}-z_{01}\right)+f_{r}\right) \end{array}$$

(6) $$\begin{array}{c} m_{2}{\ddot{z}}_{2}=-\left(k_{t_{2}}\left(z_{2}-z_{02}\right)+f_{l}\right) \end{array}$$

The mounted suspension points on both sides experience two hypothetical disturbance forces, $f_{1}$ and $f_{2}$, with equal magnitudes but opposite directions. Equations (7) and (8) can be used to explain the forces acting on the body during the vehicle’s roll and pitch motions, respectively. As shown in the following equations, namely, during roll motion, $f_{1}$ can be termed as $f_{r_{1}}$, and $f_{2}$ is comparable to $f_{r_{2}}$, whereas, during pitch motion, $f_{1}$ is equal to $f_{p_{1}}$, and $f_{2}$ is equal to $f_{p_{2}}$.

(7) $$\begin{array}{cc} f_{r_{1,2}} & =\left|F\cos\left(\theta_{s}\right)-\frac{mgh}{\left(a+b\right)}\sin\left(\theta_{s}\right)\right| \end{array}$$

(8) $$\begin{array}{cc} f_{p_{1,2}} & =\left|F-\frac{mgh}{\left(a+b\right)}\sin\left(\theta_{s}\right)\right| \end{array}$$

where the centrifugal or inertial force, *F*, occurs during cornering, vehicle acceleration, or braking. Additionally, *g* represents the gravitational force, and $\theta_{s}$ represents the slope of the road.

### 2.2. Desired Roll Angle

**Figure 2:**
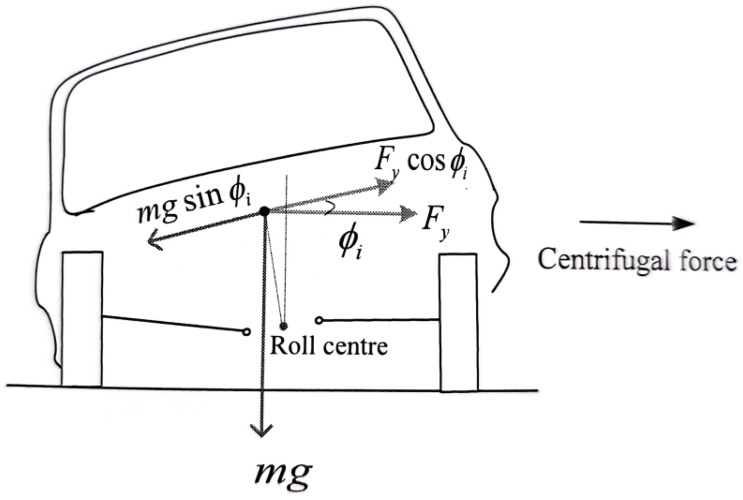
Schematic diagram for deriving the desired roll angle of a vehicle traveling at 120 km/h speed during cornering.

A schematic in Figure 2 shows how to compute the desired roll position of the vehicle body during cornering.

(9) $$\begin{array}{cc} mg\sin\left(\theta_{s}+\theta_{dr}\right) & =ma_{ca}\cos\left(\theta_{s}+\theta_{dr}\right) \end{array}$$

Rearranging the terms, we obtain:

(10) $$\begin{array}{cc} \frac{a_{ca}}{g} & =\frac{\tan\left(\theta_{dr}\right)+\tan\left(\theta_{s}\right)}{1-\tan\left(\theta_{s}\right)\tan\left(\theta_{dr}\right)} \end{array}$$

where $\theta_{dr}$ represents the desired roll angle, which can be obtained as:

(11) $$\begin{array}{cc} \theta_{dr} & =\arctan\left(\frac{a_{ca}-g\tan\left(\theta_{s}\right)}{g+a_{ca}\tan\left(\theta_{s}\right)}\right) \end{array}$$

where $a_{ca}$ is called the centrifugal acceleration.

### 2.3. Desired Pitch Angle

**Figure 3:**
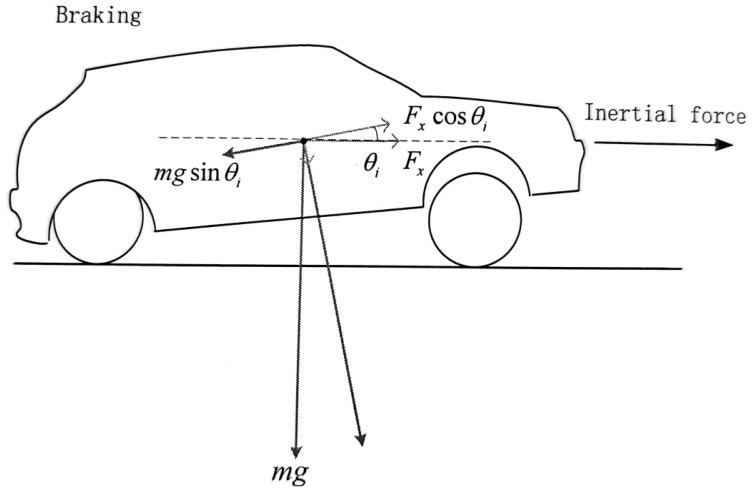
Schematic diagram for deriving the desired pitch angle of a vehicle traveling at 120 km/h speed during acceleration or braking.

Figure 3 displays the optimal location for pitch on a sloped surface. When driving on a road with slope $\theta_{s}$, the car’s body should be parallel to the horizontal axis of the road. Equations (12)–(14) describe the calculation to determine the desired pitch angle.

(12) $$\begin{array}{cc} mg\sin\left(\theta_{s}+\theta_{dp}\right) & =ma_{ia}\cos\left(\theta_{dp}\right) \end{array}$$

Rearranging the terms, we obtain:

(13) $$\begin{array}{cc} \frac{a_{ia}}{g} & =\tan\left(\theta_{dp}\right)\cos\left(\theta_{s}\right)+\sin\left(\theta_{s}\right) \end{array}$$

The desired pitch angle obtained can be given as:

(14) $$\begin{array}{cc} \theta_{dp} & =\arctan\left(\frac{a_{ia}}{g\cos\left(\theta_{s}\right)}\right)-\theta_{s} \end{array}$$

The roll angle, $\theta_{dr}$, specified in Equation (11) and the pitch angle, $\theta_{dp}$, specified in Equation (14) are calculated to counteract the external lateral and longitudinal forces that affect the passenger’s ride comfort. Throughout the vehicle’s motion, hypothetical body forces acting on both suspension mounting points have equal magnitudes and opposite directions. These forces are examined in the context of a car driving on a banked road, with particular attention paid to the magnitudes of hypothetical body forces during roll and pitch motions. To accomplish this, the jerk of control forces produced by the aerodynamic surfaces cancel out the derivative of these hypothetical body forces.

### 2.4. Aerodynamic Forces

This paper aims to improve a vehicle’s ride performance using an active aerodynamic-based anti-jerk control strategy. By smoothly operating the aerodynamic surfaces, varying downward control forces can be produced, allowing the configuration of the AAS to deal with its distribution. This distribution significantly impacts the vehicle’s ride performance, enabling adjustments to the sprung mass system vertical load to influence suspension deflection, vehicle body acceleration, and tire deflection. The AASs that generate the necessary control forces are given as follows:

(15) $$\begin{array}{c} u_{1,2}=\frac{1}{2}v^{2}\rho SC_{lift}\left(\alpha \right) \end{array}$$

The lift coefficient, $C_{lift}$, of the airfoil depends upon several variables, including the air density, ρ; vehicle speed, *v*; surface area, *S*; the angle of attack, α; shape; and surface roughness. By differentiating Equations (1) and (2), we can obtain equations of heaving and angular jerk, as given in (16) and (17), to design an anti-jerk controller with constantly known predicted information.

(16) $$\begin{array}{cc} M{\dddot{z}}_{c} & ={\dot{f}}_{l}+{\dot{f}}_{r}+{\dot{u}}_{1}+{\dot{u}}_{2}+{\dot{f}}_{1}+{\dot{f}}_{2} \end{array}$$

(17) $$\begin{array}{cc} I\dddot{\theta } & =a\left({\dot{f}}_{r}+{\dot{u}}_{1}\right)-b\left({\dot{f}}_{l}+{\dot{u}}_{2}\right)+{\dot{f}}_{1}+{\dot{f}}_{2} \end{array}$$

This study presents a novel anti-jerk predictive control approach designed to reduce the root-mean-square value of control jerk while enhancing the performance of aerodynamic surfaces. The optimal controller aims to minimize the cost function given in Equation (18), consisting of heaving and angular acceleration, suspension and tire deflection, the difference between the actual and desired attitude angle, and the jerk terms for the control inputs multiplied by the weighting constants, $\rho_{1},\rho_{2},\rho_{3},\rho_{4},\rho_{5},\rho_{7}$, respectively. These weights establish the optimal distribution of the optimized criterion’s various components.

(18) $$J=\begin{array}{c} \underset{T\to \infty }{lim}\frac{1}{2T}\int_{0}^{T}(\rho_{1}{\ddot{z_{c}}}^{2}+\rho_{2}{\ddot{\theta }}^{2}+\rho_{3}{\left(z_{c}+a\theta -z_{1}\right)}^{2}+\rho_{3}{\left(z_{c}-b\theta -z_{2}\right)}^{2}\\ +\rho_{4}{\left(\theta -\theta_{d}\right)}^{2}+\rho_{5}{\left(z_{1}-z_{01}\right)}^{2}+\rho_{6}{\left(z_{2}-z_{02}\right)}^{2}+\rho_{7}{\dot{u_{1}}}^{2}+\rho_{7}{\dot{u_{2}}}^{2})d\tau \end{array}$$

## 3. Optimal Anti-Jerk Controller Formulation

### 3.1. System Description

Continuous-time state-space model of the proposed half-car model can be represented as:

(19) $$\begin{array}{cc} \dot{x}\left(t\right) & =Ax\left(t\right)+B\dot{u}\left(t\right)+Dw\left(t\right) \end{array}$$

where x(t) represents the system’s state vector, $\dot{u}$ denotes the control jerk input, and w(t) is known as the disturbance jerk acting on the vehicle body:

$$\begin{array}{cc} x & =\left[z_{c}\;{\dot{z}}_{c}\;{\ddot{z}}_{c}\;\theta \;\dot{\theta }\;\ddot{\theta }\;z_{1}-z_{0_{1}}\;{\dot{z}}_{1}\;z_{2}-z_{0_{2}}\;{\dot{z}}_{2}\;z_{1}\;z_{2}\right] \end{array}$$

$$\dot{u}={\left[\begin{array}{cc} {\dot{u}}_{1} & {\dot{u}}_{2} \end{array}\right]}^{T},w={\left[\begin{array}{cccc} 0 & 0 & {\dot{f}}_{1} & {\dot{f}}_{2} \end{array}\right]}^{T}$$

$$\begin{array}{cc} x_{d} & =\left[0\;0\;0\;\theta_{d}\;0\;0\;0\;0\;0\;0\;0\;0\right] \end{array}$$

The constant matrices, A and B, have the appropriate dimensions, as reported in our previous work [43]. The elements of the disturbance matrix, D, are given in Appendix A.

### 3.2. Anti-Jerk Controller Design

The performance index presented in Equation (18) can be written in matrix form regarding the difference between the desired and current state, the jerk control inputs, and jerk disturbance inputs.

(20) $$J=\begin{array}{c} \underset{T\to \infty }{lim}\frac{1}{2T}\int_{0}^{T}({\left(x-x_{d}\right)}^{T}Q\left(x-x_{d}\right)+2{\left(x-x_{d}\right)}^{T}N_{2}w+2{\left(x-x_{d}\right)}^{T}N_{1}\dot{u}\\ +{\dot{u}}^{T}R\dot{u}+2w^{T}M_{1}\dot{u}+w^{T}M_{2}w)d\tau \end{array}$$

The matrices, $Q,R,N_{1},N_{2},M_{1}$, and $M_{2}$, refer to positive definite matrices, as given in Appendix A. If the pair (A,B) is assumed to be stable and (A,Q) is detectable, then the anti-jerk controller can be derived by minimizing the performance index described in Equation (20):

(21) $$\dot{u}=-R^{-1}\left(\left(B^{T}P-N_{1}^{T}\right)x+N_{1}^{T}x_{d}+M_{1}^{T}w+B^{T}g_{R}\right)$$

where *P* is the solution of the algebraic Riccati equation (ARE) given in Equation (22):

(22) $$0=Q_{n}^{T}+A_{n}^{T}P+PA_{n}-PBR^{-1}B^{T}P$$

where

$$\begin{array}{cc} A_{n} & =A+BR^{-1}N_{1}^{T}\\ Q_{n} & =Q-N_{1}R^{-1}N_{1}^{T} \end{array}$$

**Figure 4:**
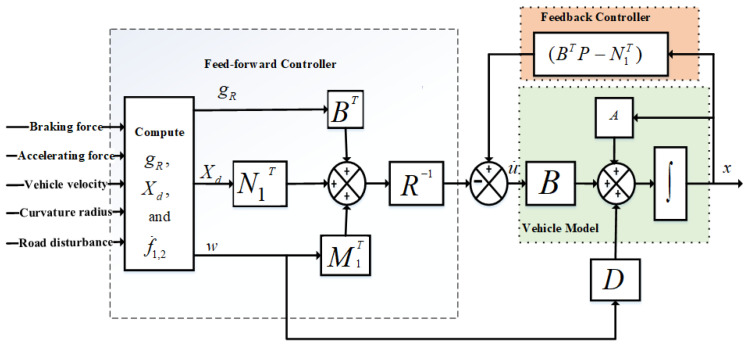
Block diagram consists of feed-forward controller to detect future road maneuvers and feedback controller to reduce tracking errors.

The block diagram for the proposed anti-jerk control strategy is shown in Figure 4. The control strategy is composed of two parts: the fee-forward part $N_{1}^{T}x_{d}+M_{1}^{T}w+B^{T}g_{R}$, to provide an anticipated action against vehicle body jerks, and the feedback part $-R^{-1}(\left(B^{T}P-N_{1}^{T}\right)x+N_{1}^{T}x_{d}$, to reduce the tracking error between actual and desired attitude angle. The vector $g_{R}$ satisfies:

(23) $$\begin{array}{c} g_{R}=\int_{0}^{tp}e^{-Ac^{T}\tau }\left(\left(PD_{n}-N_{n}\right)w\left(\tau \right)-\left(Q_{n}+PBR^{-1}N_{1}^{T}\right)x_{d}\left(\tau \right)\right)d\tau \end{array}$$

where $D_{n}=D-BR^{-1}M_{1}^{T}$, $N_{n}=N_{2}-N_{1}R^{-1}M_{1}^{T}$, and $A_{c}=A_{n}-BR^{-1}B^{T}P$ are called asymptotically closed-loop stable matrices. The closed system equation can be obtained by putting Equation (21) into (19), as given in (24):

(24) $$\begin{array}{c} \dot{x}=A_{c}x+D_{n}w-BR^{-1}NT^{1}x_{d}-BR^{-1}B^{T}g_{R} \end{array}$$

## 4. Simulation Results and Discussion

In this section, the simulation results are discussed for both the lateral and longitudinal models of the vehicle traveling at 120 Km/h, which are conducted using MATLAB 2022b installed on a Samsung Core™ 5-6400 CPU @ 2.70 GHz. The performance of the proposed anti-jerk predictive control strategy (AJPC) is evaluated by using different weighting factors, as defined in Equation (18). Table 2 shows the tuning parameters for the proposed control strategy, which are selected based on rms values considering the individual performance of heaving, rolling and pitching jerks, jerk controller, tire and suspension deflections, and total vehicle performance. A detailed explanation of how these weights can be selected is given in our previous work [43]. Different scenarios are considered to investigate the effectiveness of the proposed AJPC. In the first case, the simulations are conducted for lane-change maneuvers to evaluate the performance of the proposed AJPC to track the desired roll angle and improve ride comfort and road-holding capability. For the second case, pitch motion simulations are performed during braking and accelerating the vehicle to analyze the proposed control strategy’s impact on minimizing oscillations in the vehicle body’s vertical motion and tire deflection. The control strategy successfully mitigated the external centrifugal and longitudinal jerks acting on the vehicle body and helped to achieve the desired attitude motion. Comparing the performance of anti-jerk predictive control and predictive control (PC) without jerk using the root mean square error (RMSE) method, the AJPC outperformed the PC without jerk in terms of ride comfort and road-holding capability. The findings imply that prioritizing high weighting for control jerk can have a detrimental effect on vehicle performance. Although the impact on the desired attitude angle tracking is minor and unlikely to affect overall vehicle performance significantly, it is worth noting that this research aims not only to enhance both ride comfort and vehicle handling simultaneously but also to achieve more realistic operation of aerodynamic surfaces. Because the AASs are installed on the vehicle’s sprung mass, ride comfort is directly related to the oscillations during the motion of the vehicle’s sprung mass. Therefore, the movement of AAS directly impacts ride comfort.

### 4.1. Roll Angle Tracking

**Figure 5:**
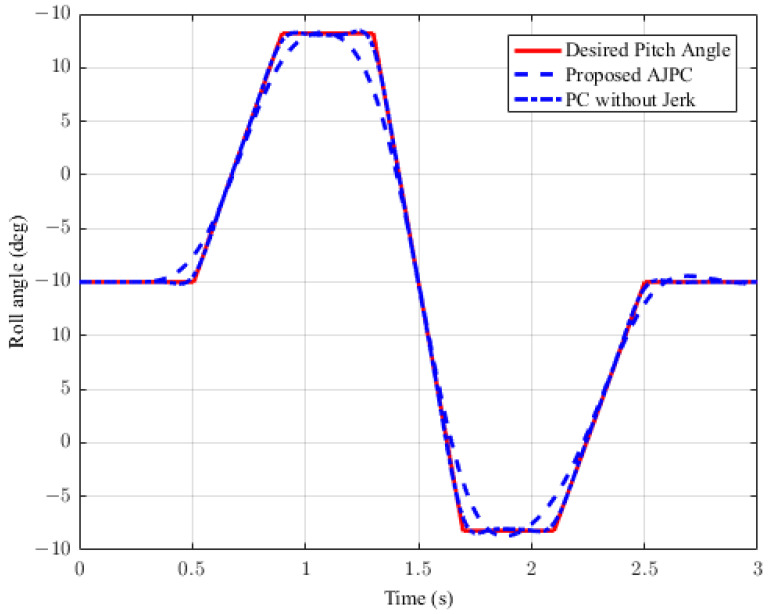
Desired roll angle tracking of a half-car model traveling during a double-lane-change maneuver at 120 km/h.

**Figure 6:**
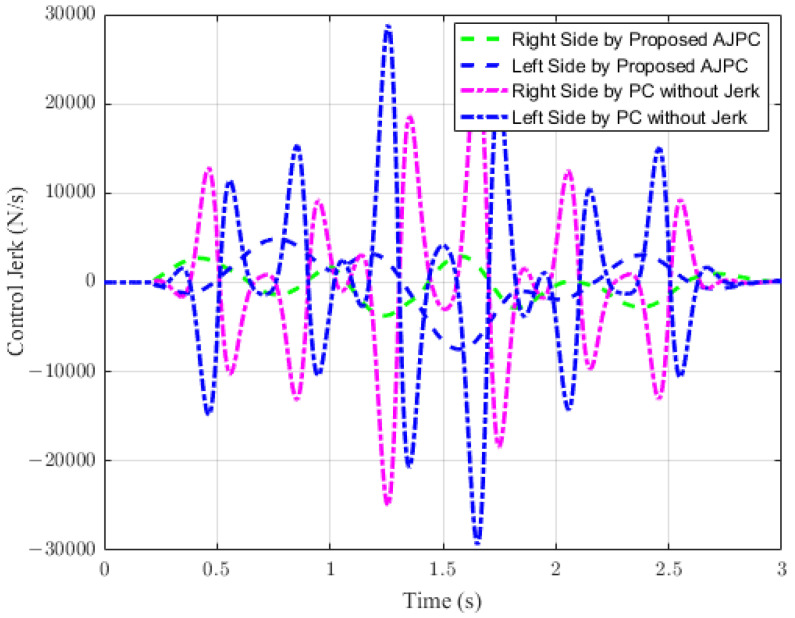
Control jerk of a vehicle traveling on a double-lane-change maneuver at 120 km/h.

**Figure 7:**
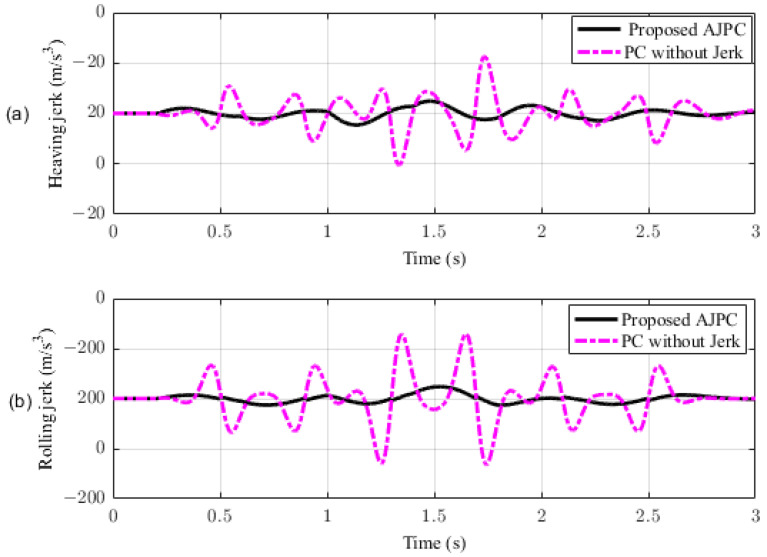
Heaving and rolling jerk of a half-car model traveling during a double-lane-change maneuver at 120 km/h. (**a**) Heaving jerk, (**b**) rolling jerk.

**Figure 8:**
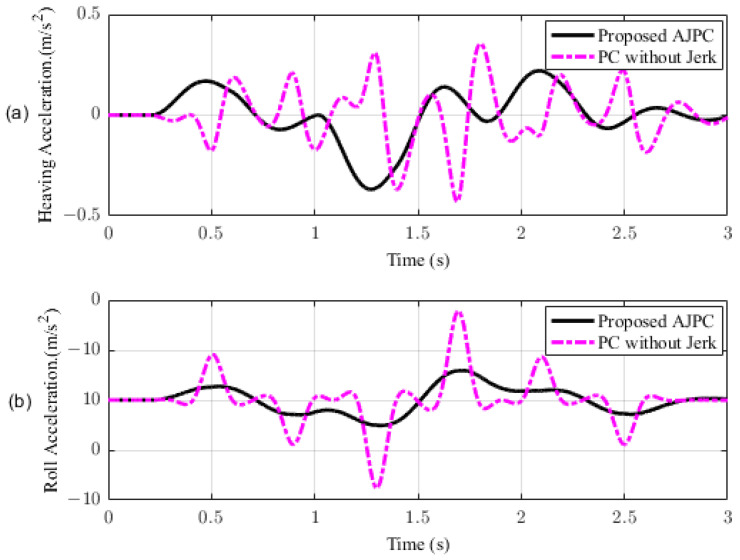
Heaving and rolling acceleration of a half-car model traveling during a double-lane-change maneuver at 120 km/h. (**a**) Heaving acceleration, (**b**) rolling acceleration.

**Figure 9:**
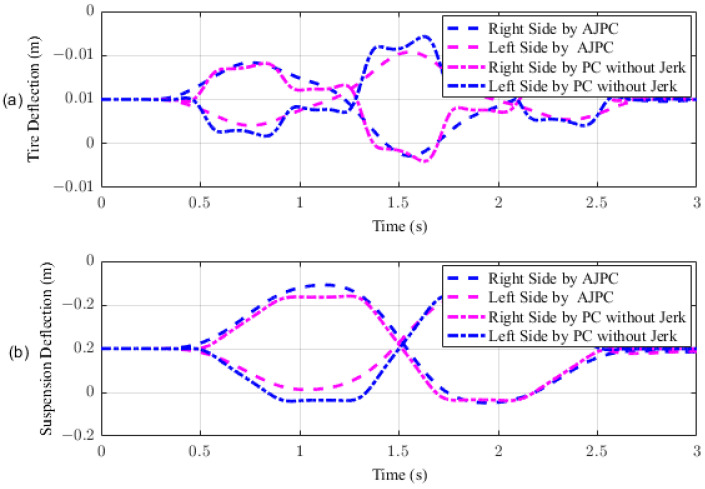
Tire and suspension deflection of half-car model traveling during a double-lane-change maneuver at 120 km/h. (**a**) Tire deflection, (**b**) suspension deflection.

This section presents the simulation results for a half-car model equipped with aerodynamic surfaces, traveling at a constant speed of 120 km/h during a double-lane-change maneuver. The performance of the proposed AJPC is investigated in the presence of centrifugal jerks acting on the vehicle body during a double-lane-change maneuver. The aim is to tilt the vehicle body inwardly during cornering to improve passenger ride comfort and road-holding capability. Figure 5 illustrates the simulation results for the desired roll angle tracking of the vehicle, which show that the tracking performance of the proposed AJPC is slower than that of the PC without jerk, mainly due to the slower motion of the aerodynamic surfaces, as shown in Figure 6. Figure 6 shows the results for the jerk control input, where the controlling jerk for the proposed AJPC has minimum overshoots, confirming that the smooth and realistic operation of the aerodynamic surfaces has a great impact on the vehicle’s ride performance. Therefore, the proposed AJPC strategy improved ride comfort while maintaining the aerodynamic surfaces’ realistic motion at the cost of slower desired attitude motion tracking. The simulation results for the vehicle body jerk are shown in Figure 7, which shows the proposed control strategy successfully reduces the heaving and rolling jerks during cornering. This can also be verified from Table 3, where, compared to the predictive control without jerk, the proposed AJPC reduces heaving jerk by 18% and rolling jerk by 21%. Hence, this confirms an improvement in ride comfort. Figure 8 shows the heaving and roll acceleration simulation results. The proposed AJPC strategy resulted in lower heaving and roll acceleration than the PC strategy without jerk. Table 3 further highlights that the proposed AJPC strategy reduced heaving acceleration by 14% and rolling acceleration by 27%, thus enhancing ride comfort. Figure 9 shows the tire and suspension deflection simulation results. The results indicate that the tire deflection causes high overshoots for PC without jerk, weakening the tire’s grip on the road while turning during a double lane-change maneuver. Table 3 indicates a 7% enhancement in road-holding capability for the proposed AJPC strategy, when compared to the PC without a jerk strategy.

### 4.2. Desired Pitch Angle Tracking

**Figure 10:**
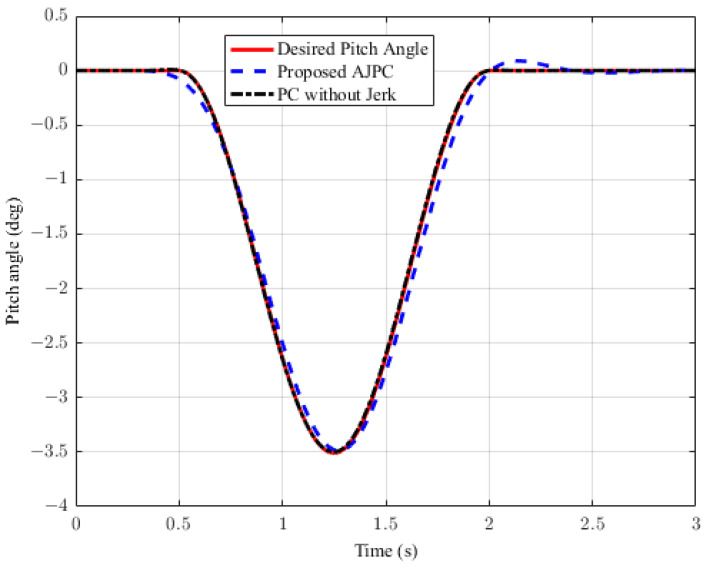
Simulation results for desired pitch angle tracking of a half-car model during acceleration.

**Figure 11:**
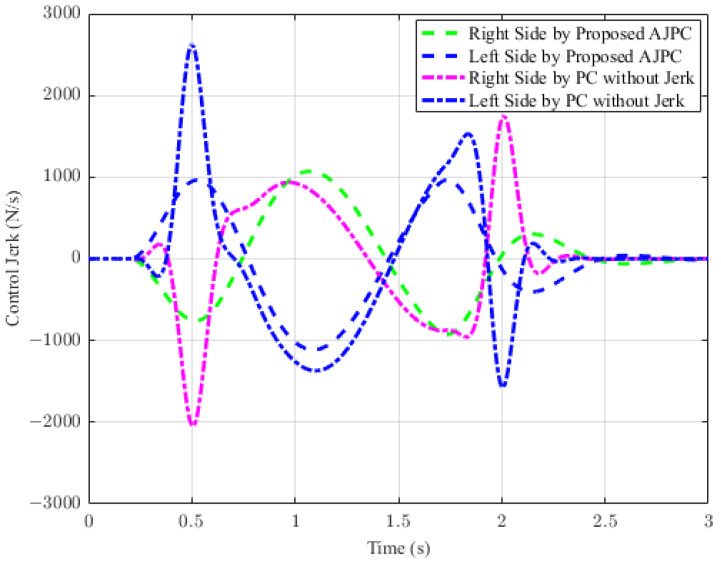
Simulation results for control jerk of a vehicle during acceleration.

**Figure 12:**
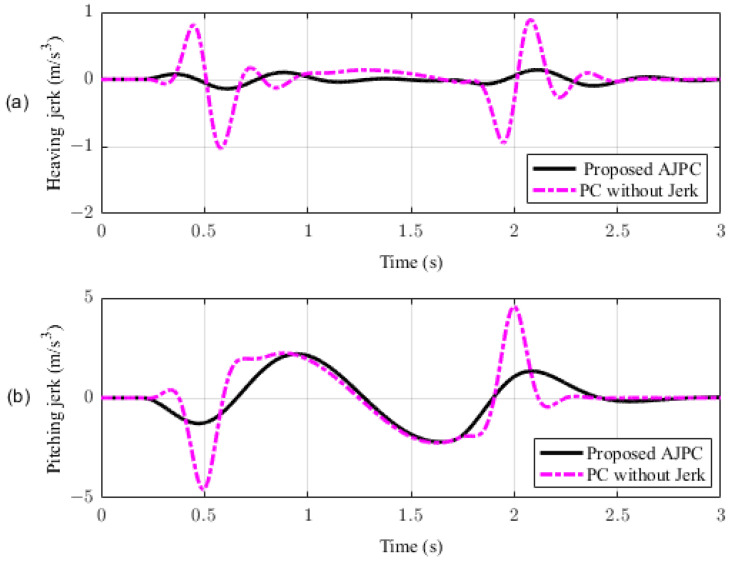
Simulation results of heaving and pitching jerks of a half-car model during acceleration. (**a**) Heaving acceleration, (**b**) pitching acceleration.

**Figure 13:**
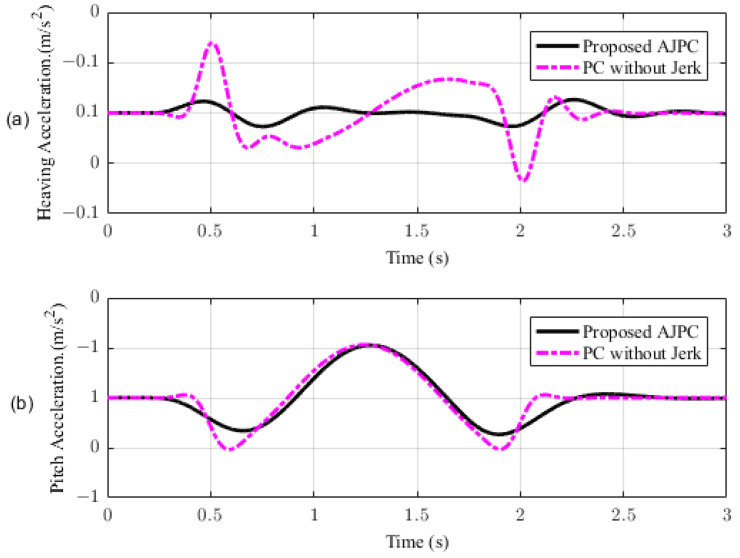
Simulation results of heaving and pitching accelerations of a half-car model during acceleration. (**a**) Heaving acceleration, (**b**) pitching acceleration.

**Figure 14:**
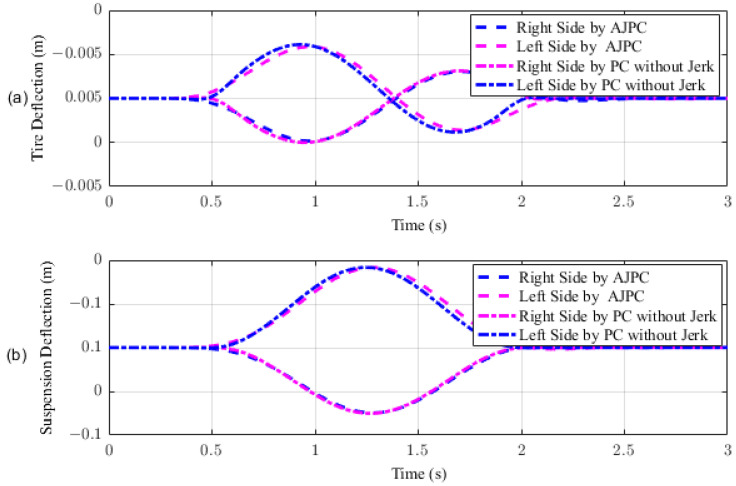
Simulation results for tire and suspension deflections of a half-car model during acceleration. (**a**) Tire deflection, (**b**) suspension deflection.

In this section, the simulation results are carried out for the half-car model when traveling at 120 km/h, considering two different cases. In the first case, the simulations are performed while accelerating the vehicle in the forward direction. The accelerating force will generate inertial forces to produce a backward pitch motion, resulting in discomfort to the passenger. The optimal solution is to adjust the vehicle’s forward pitch to cancel the inertial effects that occur while accelerating the vehicle. This will make passengers feel comfortable, with the ideal pitch angle being zero. Figure 10 displays the simulation results for desired pitch angle tracking, demonstrating that compared to the anti-jerk predictive control strategy, the predictive control strategy exhibits outstanding performance in tracking the desired pitch motion. The poor tracking by the proposed AJPC strategy is due to the realistic slower motion of the aerodynamic surfaces, as shown in Figure 11. Figure 11 shows the simulation results for control input jerk, which indicates that, compared to the PC without jerk, the proposed control strategy helps the AAS to operate smoothly to achieve minimum vehicle body jerk. This is because the operation of aerodynamic surfaces is very effective in improving the vehicle’s ride performance. For example, as shown in Figure 12, both heaving and pitching jerks are reduced in the case of AJPC, which shows that, compared to PC without jerk, the proposed AJPC strategy successfully improves ride comfort. Similarly, comparing the rms values in Table 4 indicates that the proposed AJPC strategy has 16% lower heaving and 9% lower pitching jerk. The reduction in the rms values validates a significant improvement in ride comfort. Similarly, Figure 13 shows the simulation results for heaving and pitching accelerations. This indicates that, compared to the PC without jerk, the proposed control strategy has lower heaving and pitching acceleration. This can be verified from Table 4, which shows that, in the case of the proposed AJPC strategy, heaving acceleration is reduced by 16%, and pitching acceleration is reduced by 7%. Hence, this indicates an improvement in ride comfort. Figure 14 shows the simulation results for tire and suspension deflections, which show that, while improving the ride comfort, the tire’s grip on the road is not degraded for the AJPC strategy. Table 4 also shows that both the tire and suspension deflections for the proposed AJPC strategy and PC strategy are almost identical. Hence, we can conclude that using high weights for the control jerk inputs can successfully improve ride comfort without degrading road-holding capability at the cost of slow attitude motion tracking.

**Figure 15:**
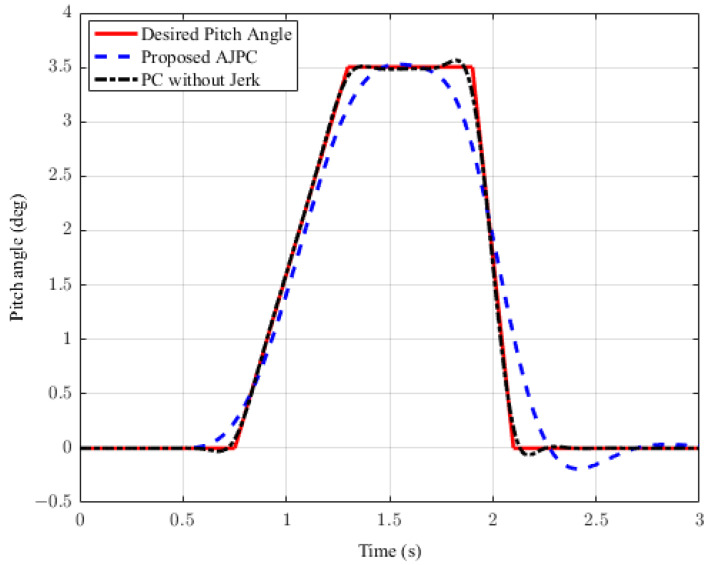
Desired pitch angle tracking of a half-car model during braking.

**Figure 16:**
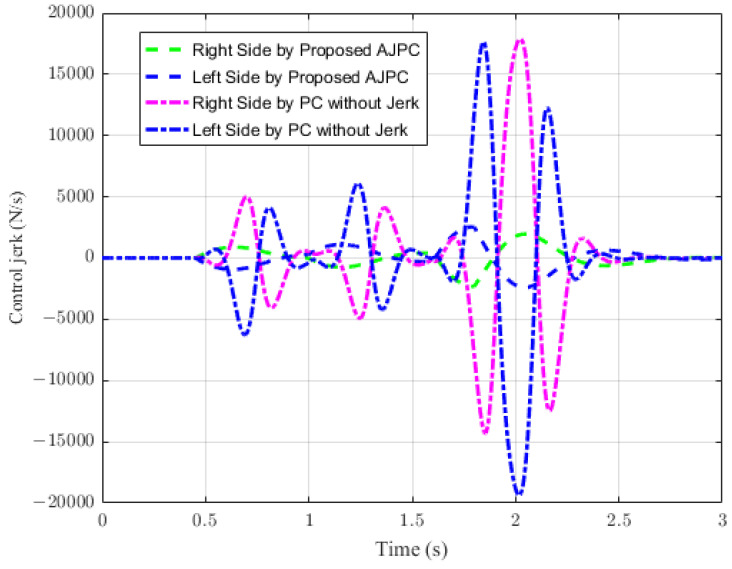
Simulation results for control jerk of a vehicle during braking.

**Figure 17:**
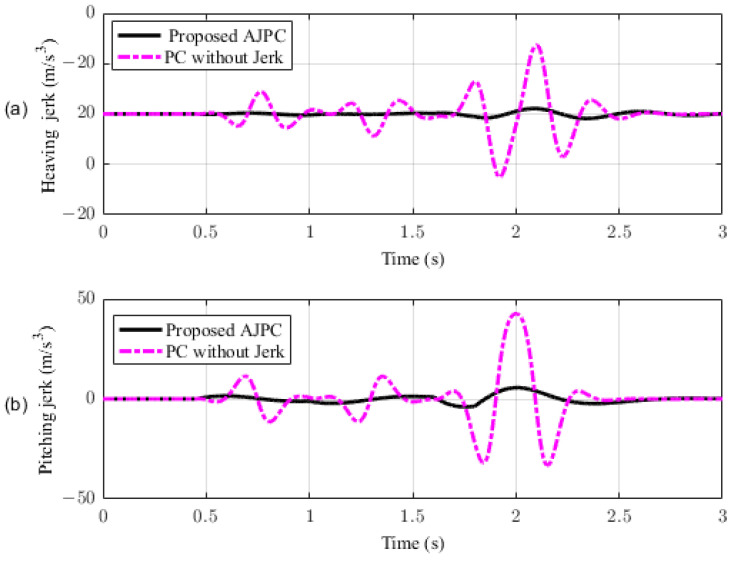
Simulation results for heaving and pitching jerks of a half-car model during braking. (**a**) Heaving jerk, (**b**) pitching jerk.

**Figure 18:**
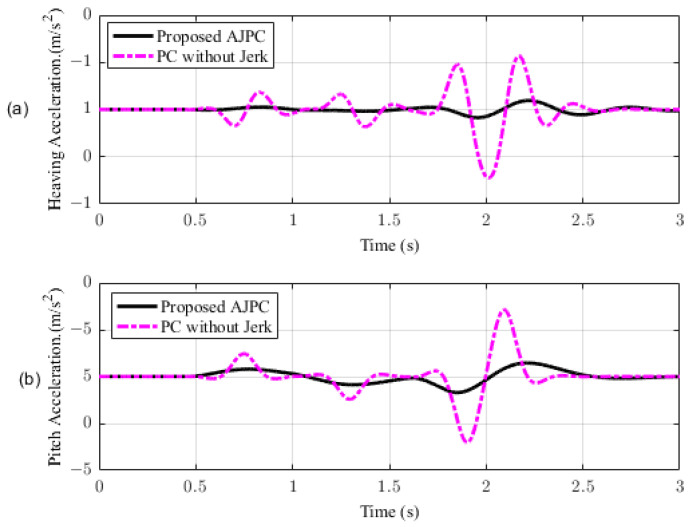
Simulation results for heaving and pitching accelerations of a half-car model during braking. (**a**) Heaving acceleration, (**b**) pitching acceleration.

**Figure 19:**
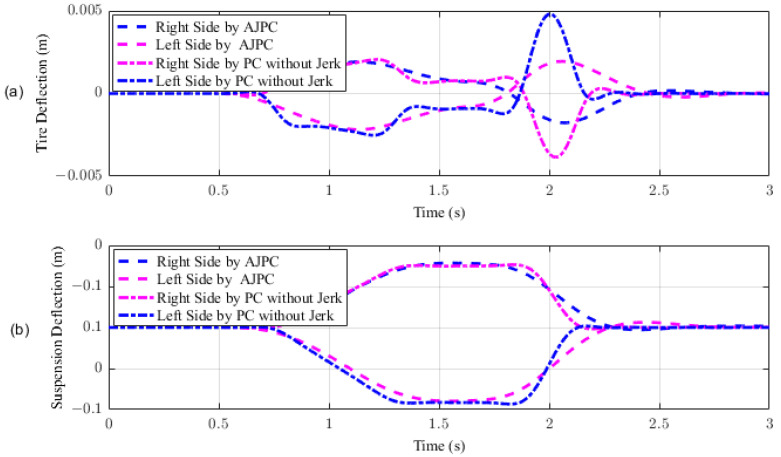
Simulation results for tire and suspension deflections of a half-car model during braking. (**a**) Tire deflection, (**b**) suspension deflection.

For the second case, the simulation results are carried out for the half-car model while braking when traveling on a sloped road. During braking, the braking force will generate inertial forces to produce a forward-pitch motion that can cause passenger discomfort. The optimal solution is to adjust the vehicle’s backward pitch to cancel out the inertial effects that occur while braking. This will allow passengers to feel comfortable with the ideal backward pitch angle. Figure 15 displays the simulation results for desired pitch angle tracking, which show that, compared to the anti-jerk predictive control approach, the predictive control without jerk performs remarkably in tracking the appropriate pitch motion during braking. The realistically slower motion of the aerodynamic surfaces leads to poor tracking by the suggested AJPC approach, as shown in Figure 16. The simulation results for the control jerk in Figure 16 validate the smooth operation of AAS for the proposed AJPC strategy. However, using aerodynamic surfaces effectively can significantly enhance a vehicle’s ride quality. This is because the smooth operation of AAS will reduce both the heaving and pitching jerks and accelerations, which are important ride comfort parameters. Figure 17 shows the simulation results for heaving and pitching jerks, indicating that the proposed AJPC successfully reduces these jerks to improve ride comfort. Comparing the rms values in Table 5 shows that the proposed AJPC approach has a 12% lower heaving jerk and a 3% lower pitching jerk. Figure 18 shows the heaving and pitching acceleration results while braking the vehicle. The rms base comparison in Table 5 indicates that, compared to the PC without jerk, heaving acceleration for the AJPC is lowered by 11%, and pitching acceleration is lowered by 13%. This confirms an improvement in ride comfort. The simulation results for tire and suspension deflections are shown in Figure 19, demonstrating that, while the AJPC method enhances ride comfort, the tire’s grip on the road is not compromised. Thus, we can conclude that the proposed AJPC successfully improves ride performance at the cost of poor pitch motion tracking.

The implementation of the proposed strategy raises the question about whether to use a force control or a discrete-time implementation of its derivative, given the availability of a force actuator; while a force control approach may be preferred, a discrete-time implementation using the difference in the actuator force, Δu, over discrete time intervals is also feasible. One advantage of the discrete-time implementation is that the output force will remain unchanged if the computer fails, avoiding the potential for an undesired force output. Instead, it will maintain the previous force value.

## 5. Conclusions

The anti-jerk predictive control technique proposed in this research was investigated to enhance the ride performance of vehicles during attitude motion while cornering, accelerating, and braking. The proposed method improved the ride comfort and road-holding ability of the vehicle fitted with active aerodynamic surfaces by canceling the centrifugal jerks during cornering and inertial jerks during acceleration and braking. The anti-jerk controller comprised two parts: a feed-forward component that uses a future road maneuver and a feedback component to address tracking errors made up of the control approach. The proposed control technique successfully achieved the realistic motion of the AAS to reduce vehicle body jerk during attitude motion. The simulation results demonstrate that the suggested technique successfully reduced control jerk to improve AAS performance and reduced heaving, rolling, and pitching jerks and accelerations to improve ride comfort without compromising road-holding capability. The results further validate that the anti-jerk predictive controller enhanced the half car’s ride comfort and stability, and significantly lessened the effect of hypothetical body jerks. The following future recommendations can be taken into account to explore the applications of the proposed approach:

- The current work can be extended to a full-car model, along with the actuator dynamics of the airfoil.
- A discrete-time implementation of the suggested control method will allow future research on the proposed control strategy.
- Robust and intelligent control algorithms may be considered to address both air and road disturbances.

## Figures and Tables

**Table 1:** Parameter values of a four-DOF half-car lateral and longitudinal model.

Symbol	Lateral Model	Longitudinal Model	Unit
*M*	500	500	kg
*I*	274	1222	kg m${}^{2}$
$m_{1},m_{2}$	25	25	kg
$k_{s_{1}},k_{s_{2}}$	10	10	kN/m
$k_{t_{1}},\;k_{t_{2}}$	1	1	kN/m
$b_{s_{1}},b_{s_{2}}$	1	1	kN/m
*a*	0.74	1.25	m
*b*	0.74	1.1	m
*h*	0.70	0.70	m

**Table 2:** The weighting factors used for the AJPC and PC in performance indices.

Weighting Factors	Targets	Lateral Model		Longitudinal Model	
		AJPC	PC	AJPC	B
$\rho_{1}$	Heaving acceleration	1	1	1	1
$\rho_{2}$	Angular acceleration	1	1	1	1
$\rho_{3}$	Suspension deflection	$10^{4}$	$10^{4}$	$10^{4}$	$10^{4}$
$\rho_{4}$	Angle tracking	$10^{9}$	$10^{9}$	$10^{9}$	$10^{9}$
$\rho_{5}$	Tire deflection	$2\times 10^{5}$	$2\times 10^{5}$	$10^{7}$	$10^{7}$
$\rho_{7}$	Jerk controller	$10^{-2}$	$10^{-4}$	$10^{-2}$	$10^{-4}$

**Table 3:** Root mean square error (RMSE) values for roll motion.

Parameter	PC without Jerk	AJPC
Heaving jerk	100	82.11
Rolling jerk	100	79.23
Jerk controller	100	40.25
Heaving acceleration	100	86.38
Roll acceleration	100	73.12
Tire deflection	100	92.67
Suspension deflection	100	99.83

**Table 4:** Root mean square error (RMSE) values for pitch motion during accelerating.

Parameter	PC without Jerk	AJPC
Heaving jerk	100	84.10
Pitching jerk	100	91.31
Jerk controller	100	63.32
Heaving acceleration	100	85.80
Pitching acceleration	100	93.78
Tyre deflection	100	99
Suspension deflection	100	100

**Table 5:** Root mean square error (RMSE) values for pitch motion during braking.

Parameter	PC without Jerk	AJPC
Heaving jerk	100	88.40
Pitching jerk	100	85.14
Jerk controller	100	45.11
Heaving acceleration	100	89.72
Pitching acceleration	100	87.78
Tire deflection	100	98.54
Suspension deflection	100	90.32

## Data Availability

Not applicable.

## Nomenclature

The following are the mathematical notions used in this paper:

*M*	Vehicle body sprung mass
$m_{1},m_{2}$	Unsprung masses
*I*	Moment of inertia
$k_{s_{1}},k_{s_{2}}$	Spring stiffness coefficients of right and left side or front and rear sides
$k_{t_{1}},k_{t_{2}}$	Tire stiffness coefficients of right and left side or front and rear sides
$b_{s_{1}},b_{s_{2}}$	Damping coefficients of right and left side or front and rear sides
*a*	Distance of the right or front side from the center of mass
*b*	Distance of the left or rear side from the center of mass
*h*	Height of the center of mass from the ground
$z_{c}$	Sprung mass displacement
θ	Roll or pitch angle of the vehicle body
$\theta_{s}$	Road slope
*g*	Gravitational acceleration
$a_{ca}$	Centripetal acceleration
$a_{ia}$	Inertial acceleration
$d_{r}$	Desired roll angle
$d_{p}$	Desired pitch angle
$z_{1},z_{2}$	Unsprung mass displacements of right and left or front and rear sides
$z_{01},z_{02}$	Road disturbances at right and left or front and rear sides
$f_{1}$	Hypothetical body force acting at the right or front side of the vehicle body
$f_{2}$	Hypothetical body force acting at left or rear side of the vehicle body
*v*	Vehicle speed
$\dot{w}$	Disturbance input
*F*	Centrifugal force during cornering or inertial force during accelerating of braking
ρ	Air density
*S*	Surface area of the airfoil
α	Angle of attack of the airfoil
$C_{lift}$	Lift coefficient of the airfoil
$\rho_{1}$	Weighing constant for heaving acceleration
$\rho_{2}$	Weighing constant for angular acceleration
$\rho_{3}$	Weighing constant for suspension deflection
$\rho_{4}$	Weighing constant for desired angle tracking
$\rho_{5}$	Weighing constant for tire deflection
$\rho_{7}$	Weighing constant for jerk controller

## Appendix A

The non-zero entries for matrix D are

$$\begin{array}{c} d_{33}=d_{34}=1/M\\ d_{63}=a/I,d_{64}=-b/I \end{array}$$

The elements of the weight matrix, Q, defined in (20) are given as follows:

$$q_{ij}=\left\{\begin{array}{cc} \rho_{1}a_{3i}a_{3j}+\rho_{2}a_{6i}a_{6j} & \;\mathrm{if}\;i\neq j\\ \psi_{ij} & \;\mathrm{if}\;i=j \end{array}\right.$$

with special entries as:

$$\begin{array}{c} \psi_{11}=\rho_{1}a_{31}a_{31}+\rho_{2}a_{61}a_{61}+\rho_{3},\\ \psi_{33}=\rho_{1}a_{33}a_{33}+\rho_{2}a_{63}a_{63}+\rho_{1},\\ \psi_{44}=\rho_{1}a_{34}a_{34}+\rho_{2}a_{64}a_{64}+a\rho_{3}-b\rho_{3}+\rho_{4},\\ \psi_{66}=\rho_{1}a_{66}a_{66}+\rho_{2}a_{66}a_{66}+\rho_{2},\\ \psi_{77}=\rho_{1}a_{37}a_{37}+\rho_{2}a_{67}a_{67}+\rho_{5},\\ \psi_{99}=\rho_{1}a_{39}a_{39}+\rho_{2}a_{69}a_{69}+\rho_{5},\\ \psi_{1010}=\rho_{1}a_{310}a_{310}+\rho_{2}a_{610}a_{610},\\ \psi_{1111}=\rho_{1}a_{311}a_{311}+\rho_{2}a_{611}a_{611}+\rho_{3},\\ \psi_{1212}=\rho_{1}a_{312}a_{312}+\rho_{2}a_{612}a_{612}+\rho_{3} \end{array}$$

Non-zero elements of the matrices, $N_{1},N_{2},M_{1}$, and $M_{2}$, are given as follows:

$$\begin{array}{c} n_{1ij}=\rho_{1}a_{3i}b_{3j}+\rho_{2}a_{6i}b_{6j}\;i=1,\ldots ,12,\;j=1,2\\ n_{2ij}=\rho_{1}a_{3i}d_{3j}+\rho_{2}a_{6i}d_{6j}\;i=1,\ldots ,12,\;j=1,2\\ m_{1ij}=\rho_{1}d_{7i}b_{3j}+\rho_{2}d_{9i}b_{6j}\;i=1,\;j=1,2\\ m_{2ij}=\rho_{1}d_{7i}b_{3j}+\rho_{2}d_{9i}b_{6j}\;i,j=1,2 \end{array}$$
